# A Brief, Affordable, Broad-Access Transdiagnostic Intervention (Project RE-THINK) for Adolescents: Quasi-Experimental Study

**DOI:** 10.2196/65491

**Published:** 2025-12-11

**Authors:** Christopher S Rozek, Maegan B Arney, Benjamin Kedl, Jenalee R Doom, David C Rozek

**Affiliations:** 1 Department of Education, Washington University in St Louis, 1 Brookings Drive, St Louis, MO, 63130, United States, 1 314-935-6791; 2 Department of Psychology, University of Denver, Denver, CO, United States; 3 Department of Psychiatry and Behavioral Sciences, University of Texas Health Science Center San Antonio, San Antonio, TX, United States

**Keywords:** single-session interventions, scalable, transdiagnostic intervention, online intervention, school-based intervention, transdiagnostic, diagnostic, intervention, adolescent, quasi-experimental, school, youth, teenager, mental health, negative cognitions, emotion, emotional well-being, emotional outcomes, health intervention, digital mental health, adolescent mental health

## Abstract

**Background:**

Adolescence is a crucial developmental period characterized by elevated stress and significant mental health challenges, including depression and anxiety. With barriers, such as stigma, accessibility, and cost hindering effective treatment, leveraging school systems for mental health interventions offers a strategic advantage due to their reach and potential for scalability.

**Objective:**

This study aimed to investigate the immediate impact of “Project RE-THINK,” a single-session, digital thought record intervention delivered in a school setting, on negative cognitions and overall emotional valence among adolescents.

**Methods:**

Project RE-THINK helps adolescents to identify, examine, and challenge negative cognitions to improve their mental health, as demonstrated through changes in negative cognition and overall emotional valence. Adolescents (N=1052) in grades 10‐12 enrolled in high school during the 2023‐2024 school year completed the digital thought record intervention activity. Using a quasi-experimental pre/post design, participants read through an example thought record and completed their own thought record, which involved identifying and describing a recent upsetting situation, answering a series of questions to challenge their negative cognition, and learning and using emotion regulation skills regarding the upsetting situation. Measures of pre- and postintervention overall emotional valence and negative cognition were collected to determine the intervention effect on participants’ mental health.

**Results:**

Descriptive statistics confirmed that smaller proportions of adolescents endorsed feeling negative emotions, such as anger, shame, anxiety, disgust, guilt, sadness, and fear, after the intervention. Paired samples *t* tests showed that adolescents experienced a significant reduction in their belief in their negative cognition from pre- to postintervention (*t*_1051_=27.71; *P*<.001; *d*=0.85, 95% CI 0.78-0.93), which demonstrates that the intervention helped them challenge their negative thoughts about their upsetting situation, as well as significant improvements to their overall emotional valence (*t*_1051_=−31.85; *P*<.001; *d*=−0.98, 95% CI −1.06 to −0.91), which demonstrates that the intervention helped them feel better about their upsetting situation. Findings also showed a significant correlation between change in negative cognition and change in overall emotional valence (*r*=0.25; *P*<.001), supporting our hypothesis that reducing the strength of belief in negative cognitions can help improve one’s emotions. Finally, analysis of covariance (ANCOVAs) confirmed that there were no significant differences in intervention efficacy by gender, race and ethnicity, or socioeconomic status, suggesting broad intervention efficacy across adolescents from different backgrounds and experiences.

**Conclusions:**

Project RE-THINK effectively improved both cognitive and emotional outcomes among adolescents, demonstrating its potential as a scalable, low-cost intervention within school settings. Future studies should explore the longitudinal effects and potential integration of such interventions into regular school curricula to help adolescents learn effective emotions and coping skills as well as to help protect and sustain adolescent mental health.

## Introduction

### Background

Adolescence is a pivotal developmental and transitional stage marked by many social, biological, and psychological changes. These transitions increase vulnerability to mental health challenges, including heightened risks for depression and anxiety [1-5]. This vulnerability has been further exacerbated by global factors, such as the COVID-19 pandemic, which has added additional difficulties for many adolescents [6-9]. In the United States, approximately 17% of adolescents experience at least one major depressive episode annually, and around 32% report symptoms of anxiety disorders [10,11]. These numbers highlight the urgent need for accessible mental health interventions targeting adolescents.

In light of this complex landscape, having easy access to prevention and intervention programs is essential to help support individuals during this critical developmental period. Depression and anxiety are two of the most common mental health conditions to emerge during adolescence [12]. Over the past decade, estimates suggest that rates of depression have nearly doubled, with similar increases for anxiety, including higher rates in females compared to males [13]. Equally important, subclinical symptoms of mood disorders are also increasing and are associated with a heightened risk of future diagnoses [14]. The consequences of unaddressed mental health difficulties during early adolescence resonate throughout life, contributing to lower educational attainment, an increased likelihood of engaging in health risk behaviors, and death by suicide [15-18].

Cognitive behavioral therapy (CBT) is a frontline treatment for anxiety and depression and has demonstrated strong empirical support [19]. CBT centers on the interconnectedness of thoughts (or cognitions), behaviors, emotions, and physiology [20]. A core component within CBT is cognitive restructuring, which helps individuals recognize and reframe negative thought patterns [21-23]. Cognitive restructuring is particularly effective across a range of mental health conditions, making it a valuable transdiagnostic tool [21-24].

One widely used cognitive restructuring exercise is a thought record [25-27]. This exercise guides individuals through the process of evaluating what happens when an upsetting or stressful situation occurs and encourages them to identify and challenge negatively biased thoughts by examining the evidence for and against those thoughts from an unbiased perspective. The goal is to generate more balanced, realistic, and adaptive cognitions, which can lead to reductions in negative emotions and foster healthier emotional and behavioral responses [25-27]. Although thought records can differ in their style across varying CBT manuals, the core concepts are to identify the negative cognition, examine and challenge the negative cognition (eg, what is the evidence for and against the negative thought from an unbiased view), and generate a more balanced and useful thought that may be linked to reductions in negative emotions and potentially more positive emotions [25]. Although traditionally practiced over multiple therapy sessions, research indicates that thought records can yield measurable benefits even when delivered in a single session [28]. Given the link between changing negative cognitions and improving emotional well-being, CBT interventions like thought records can be used both as a preventive and intervention strategy [21-24,29].

Despite the effectiveness of CBT treatment and prevention programs, traditional delivery methods—often involving weekly sessions over several months [23,27]—can be challenging for adolescents. Barriers include stigma, limited awareness of available services, concerns about confidentiality, financial constraints, and logistical issues such as transportation and access to clinicians [30]. The substantial time commitment required (eg, 12‐20 weekly sessions) also contributes to high dropout rates, limiting the accessibility and effectiveness of these interventions [31,32]. Schools offer a unique and accessible setting for delivering mental health interventions, effectively addressing many logistical barriers faced by adolescents [32,33]. Incorporating mental health programs into school environments not only enhances psychological well-being but can also lead to improved academic outcomes, as mental health and academic success are closely linked [34]. Furthermore, school-based interventions can foster stronger, healthier relationships between students, teachers, and counselors, creating a supportive environment for adolescent mental health [35,36].

However, even within school settings, traditional resource-intensive interventions may remain challenging to implement due to time constraints and competing academic priorities. To address these challenges, single-session interventions (SSIs) have emerged as a promising, cost-effective, and scalable solution for the mental health concerns of students and overcome many of the common barriers to interventions [36-39]. Digital SSIs, particularly self-guided programs that do not require a therapist or coach involvement, offer the promise of cost-effectiveness and reduced time and resource requirements compared to traditional face-to-face therapist-delivered interventions [39]. Numerous large-scale studies have indicated that SSIs can yield meaningful improvements in crucial mental health outcomes [39-42].

### Objective

The goal of this study was to test a newly developed school-based digital thought record SSI, called Project RE-THINK (Remember to Evaluate your thoughts: Are they True, Helpful, Inconsistent, Narrow-minded, or Knowledge-based?), among adolescents. Thought records are a flexible skill applicable regardless of prior psychopathology and have been used effectively in therapy, self-help, and experimental contexts [25-27]. Thought records allow individuals to address any upsetting situation associated with an emotional response, providing flexibility for adolescents to reflect on circumstances relevant to their personal experiences rather than focus on any one particular type of scenario. Project RE-THINK was designed to function as both a prevention and intervention tool within a school setting. As a preventive measure, it equips adolescents with emotion regulation strategies that can be applied in future stressful situations, potentially reducing the likelihood of developing maladaptive coping mechanisms or more severe mental health challenges. Simultaneously, the intervention allows participants to actively engage with a currently distressing experience by applying cognitive restructuring techniques in real-time. Project RE-THINK can be used as a tool regardless of whether the student has a current mental health diagnosis. This study focuses on more immediate intervention outcomes, but the broader goal of this intervention includes the prevention of the development of serious mental health difficulties that occur over longer periods of time via the growth of emotion regulation skills taught by the activity.

Although previous research has shown that SSIs can be effective in improving mental health outcomes, larger-scale studies using a variety of SSIs are needed to build support for different interventions and how they impact various groups and outcomes [38-41]. This study examined the immediate effects of Project RE-THINK on adolescents’ negative cognitions and emotional valence, which is defined as the degree to which an individual is experiencing positive or negative emotions generally, in a school setting. The study had the following hypotheses: (1) by completing Project RE-THINK, adolescents’ strength of belief in their negative cognition will decrease and overall positive emotion valence will improve (from negative to positive) from pre- to postintervention, (2) change in strength of belief in negative cognition will be correlated with the change in overall emotional valence, such that reducing one’s negative cognition belief will be associated with a change in emotions from more negative to positive, and (3) exploratory analyses will be used to examine how adolescent demographic characteristics, including gender identity, race or ethnicity, socioeconomic status (SES), and grade level, impact the efficacy of the intervention for improving both negative cognition belief and overall emotion valence. In addition, we will examine whether the number of words written during the intervention predicts changes in negative cognition belief and overall emotion valence. No differences in these analyses would provide initial evidence suggesting the potential for broad accessibility and efficacy of this intervention across adolescent demographic groups and regardless of whether participants wrote long, detailed responses or shorter responses to the intervention prompts.

## Method

### Design and Recruitment

This study used a quasi-experimental pre/post design to assess the effects of a digital SSI on adolescents’ negative cognitions and emotional valence. While not randomized, this design allowed us to evaluate within-subject changes in cognitive and emotional outcomes following the intervention. Adolescents (N=1052) attending a Midwestern United States high school completed the thought record activity as part of their social-emotional learning curriculum assigned in their classes as normal classwork. Because of the personal nature of the activity, adolescents could skip questions without penalty.

Adolescent demographic information demonstrated both racial and ethnic and gender diversity: 53% White, 16% Hispanic or Latino, 15% Black or African American, 7% Asian, and 7% identifying in other groups (eg, Native American or Alaska Native, Native Hawaiian or other Pacific Islander, or 2 or more races), and 2% not reported; 53% female-identifying, 43% male-identifying, 3% nonbinary or not listed gender identity, and 2% not reported. We used free or reduced lunch status as a proxy for family economic resources and SES, with 75% of adolescents being from middle- or higher-income backgrounds, 23% of adolescents from lower-income backgrounds, and 2% not reported. Participants included students from tenth (36%), eleventh (36%), and twelfth grades (28%), which typically correspond to ages 15‐18 years.

### Procedure

Adolescents completed a digital intervention class activity during the 2023‐2024 school year as part of a school-wide activity in support of their well-being. The activity was self-guided and developed for an adolescent version of the thought record, a tool used in CBT to help disrupt negative cognitions and promote mental health [25,26]. Adolescents were first given the opportunity to read through an example activity to understand the organization and expected responses within the thought record activity. The thought record activity involved first identifying a situation that made the adolescent upset within the past 2 weeks. Specifically, adolescents were asked: “Describe a situation that made you feel upset in the past 2-weeks.” If adolescents were unable to identify a time when they were upset in the last 2 weeks, then they were advised to think about a situation that happened to them longer ago or think about a situation that happened to a friend or family member. Adolescents then described the situation that made them upset. Next, adolescents answered some preintervention measures about the upsetting situation, including pretask overall emotional valence, pretask individual emotions and intensity ratings, and pretask negative cognition (refer to the “Measures” section for more information on these questions).

After reporting on the upsetting situation, their emotions about it, and their negative cognition, adolescents then completed a series of questions meant to challenge their negative cognition, teach them emotion regulation skills, and improve how they felt about the upsetting situation. First, adolescents were asked to describe any evidence that might support this negative cognition as well as any evidence that might not support this negative thought with the following questions: “What is the evidence supporting your negative thought?” and “What is the evidence against your negative thought?” Next, adolescents were asked to think about their problem from an outside perspective with the question: “What would you tell your friend if they were having this thought?” Adolescents were then asked to consider and reflect on a variety of potential scenarios regarding the upsetting situation with the following questions: “What is the worst-case scenario and how could I manage it?” “What is the best-case scenario?” and “What is the most realistic-case scenario?” Finally, adolescents were asked to describe a more realistic way of thinking about the upsetting situation and think about the future: “What is a more realistic way of thinking? What can I tell myself in the future?” After writing down these more realistic approaches to thinking about the upsetting situation, adolescents rated the extent to which they believed this new thought from 0% to 100%: “How much do you believe this new thought?”

**Figure 1.**
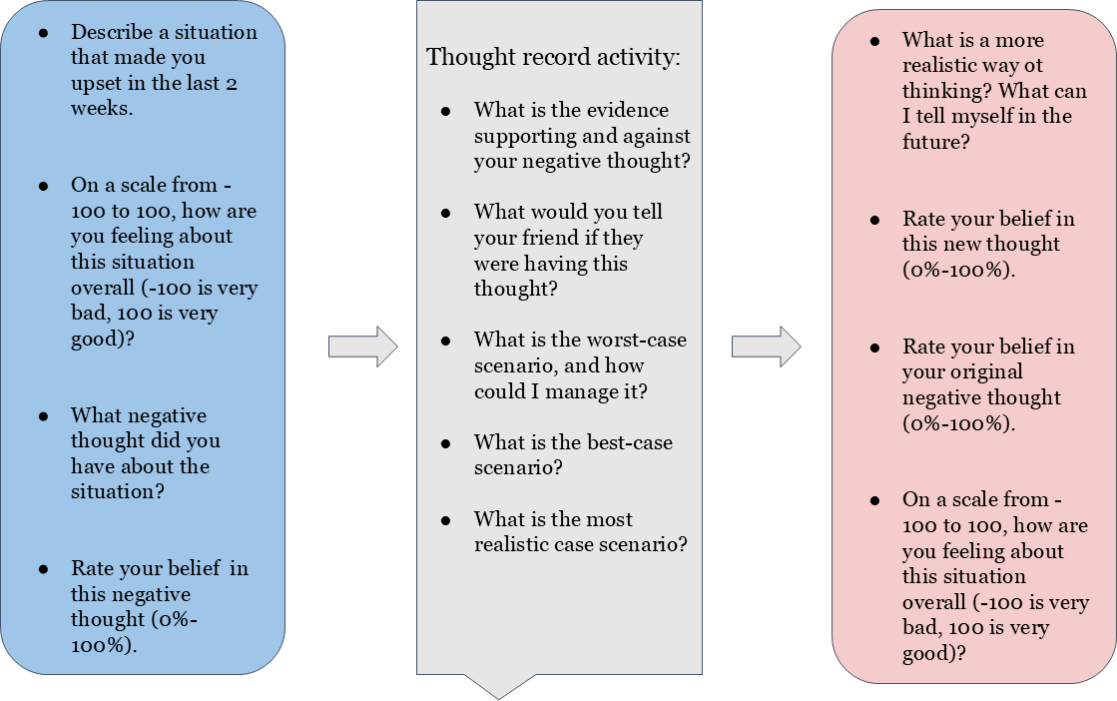
Thought record activity procedure. The thought record activity involves describing an upsetting situation, identifying associated negative cognitions and emotions, responding to prompts that help in challenging those negative thoughts, and then rerating your negative cognitions and emotions.

After completing the questions to challenge their negative thoughts, adolescents were given posttask measures to assess how they were now feeling about the upsetting situation, including posttask belief in their original negative cognition, posttask overall emotional valence, and posttask individual emotions and intensities. The individual emotions and intensities are part of the thought record task and not necessarily meant to be used as outcomes. Instead, they help adolescents work through their emotions. Pretask, adolescents are only asked to choose from negative emotions so they can focus on the upsetting nature of the situation, but post task, adolescents are asked to choose from both positive and negative emotions to help consider how they might feel more positively after the task. Thus, these measures are part of what helps adolescents feel better during the task rather than outcomes that are measured similarly both pre- and posttask. The school provided all typical accommodations to students who usually received accommodations for other similar assignments in this classroom setting for the Project RE-THINK activity, including extra time and assistance. Refer to Figure 1 for a summary of the thought record activity.

### Measures

#### Pre- and Postintervention Negative Cognition

Adolescents were asked to rate their belief in the negative cognition (or thought) that they wrote down at the beginning of the activity [25,26]. Specifically, before completing the thought record task, they were asked: “How much do you believe this thought (from 0% to 100%)?” and after completing the thought record task, they were asked: “How much do you believe your original negative thought?” Response options were on a scale from 0% to 100%.

#### Pre- and Postintervention Overall Emotional Valence

To assess overall emotional valence, we asked adolescents a pre- and posttask question to determine their general feelings about the upsetting situation they were focused on during the activity. Specifically, both before and after completing the thought record task, they were asked: “On a scale from −100 to 100, how are you feeling about this situation overall? (−100 is very bad, 100 is very Good)” [25,26]. Response options were on a sliding scale from −100 to 100.

### Analytic Plan

We analyzed the data in 3 stages using SPSS (version 29; IBM Corp). First, we reported descriptive data (eg, zero-order correlations, means and SDs of main study variables, and the choices adolescents made during the activity). Second, we tested our main research questions regarding pre/post intervention changes in negative cognition and overall emotional valence with paired samples *t* tests. Third, we investigated whether there were group differences in intervention efficacy, including adolescent demographics (ie, gender, race or ethnicity, and grade level) or the length of their written responses, as predictors of the effectiveness of the intervention. For example, we tested whether the intervention was more, less, or similarly effective for older versus younger adolescents. This analysis involved running ANCOVAs to examine group differences on postmeasures while controlling for preintervention measures (eg, controlling for preintervention measures of negative cognitions or overall emotional valence when testing for differences on postmeasures on negative cognitions or overall emotional valence).

### Ethical Considerations

As part of our research-practice partnership between researchers and the school, before the survey, adolescents chose whether or not to assent to allow researchers access to their responses after reading through an online assent form. Parents and caregivers were emailed information outlining the topics covered in the activity and were also given the option to opt out of sharing their child’s data with researchers. All study procedures were approved by the Institutional Review Board at the University of Denver, which served as a central Institutional Review Board for this multi-institution project.

## Results

### Descriptive Statistics

Basic zero-order correlations and descriptive statistics of the main study variables are included in Table 1. Pre- and postintervention measures of negative cognition and overall emotional valence were significantly correlated with each other, but word count was not significantly correlated with pre- and postmeasures of negative cognitions and overall emotional valence. Adolescents wrote an average of 91.69 words when challenging their negative cognitions.

**Table 1.** Zero-order correlations and descriptive statistics for main study variables*.* Preintervention measures were assessed before completing the thought record, and postmeasures were completed after the thought record. Word count includes all the words adolescents wrote for the thought record questions involved in challenging their negative thoughts. Higher scores on negative cognition (ranging from 0 to 100) correspond to a stronger belief in the negative cognition, and higher scores on overall emotional valence (ranging from –100 to +100) correspond to more positive emotions.

Variable	Pre negative cognition	Post negative cognition	Pre overall emotional valence	Post overall emotional valence	Word count for thought challenge responses	Mean (SD)
Prenegative cognition	1.00	—^a^	—^a^	—^a^	—^a^	67.17 (26.21)
Postnegative cognition	.57^b^	1.00	—	—	—	44.15 (31.25)
Preoverall emotional valence	–.20^b^	–.16^b^	1.00	—	—	−28.09 (46.67)
Postoverall emotional valence	–.23^b^	–.40^b^	.46^b^	1.00	—	20.98 (49.23)
Word count for thought challenge responses	–.01	–.02	.06	.01	1.00	91.69 (54.92)

a Not applicable.

b *P*<.001.

Before and after the intervention, adolescents also chose from a list of individual emotions and rated the intensity they felt for each emotion. These choices are set up in a way to be a part of the intervention activity rather than an outcome, as they initially focused on negative emotions and then included either or both positive and negative emotions at the end. The following percentages represent the proportion of adolescents who chose these emotions pre- and postintervention (preintervention percentages listed first and postintervention percentages second): 60% and 13% for anger, 30% and 9% for ashamed, 49% and 22% for anxious, 10% and 4% for disgusted, 18% and 6% for guilty, 49% and 14% for sad, and 19% and 6% for scared. In all cases, descriptively, smaller proportions of adolescents chose negative emotions after the intervention. Post intervention, emotion choices also included positive emotions, and the following percentages represent the proportion of adolescents who chose positive emotions postintervention: 17% for happy, 46% for hopeful, and 12% for proud. Adolescents could also choose “other” for their emotion and write in a response. As noted in the “Method” section, these emotion choices were intended to promote growth and positive change; thus, they are considered part of the treatment rather than an outcome, so we describe adolescent emotion choice responses here, but we do not analyze these choices.

### Main Analyses

To assess the efficacy of the intervention, we tested whether adolescents significantly reduced their belief in their negative cognitions and whether adolescents improved their overall emotional valence by testing for change from preintervention measures to postintervention. We note that there are different ways to calculate effect sizes for paired samples *t* tests, and we calculated the standardizer with the SD of the difference. Adolescents significantly reduced their belief in their negative cognition from pre- to postintervention (*t*_1051_=27.71; *P*<.001; *d*=0.85, 95% CI 0.78-0.93), suggesting that the intervention successfully helped adolescents challenge their negative cognitions. Specifically, adolescents’ strength of belief in their negative thought averaged 67.17 (SD 26.21) before the intervention and 44.15 (SD 31.25) post intervention. Adolescents significantly improved their overall emotional valence from pre- to postintervention (*t*_1051_=−31.85; *P*<.001; *d*=−0.98, 95% CI −1.06 to −0.91), suggesting that the intervention successfully helped adolescents feel better about their upsetting situation. On a −100 (very bad) to +100 (very good) scale, adolescents tended to feel negatively about their upsetting situation before the intervention (mean −28.09, SD 46.67) but felt positively after the intervention (mean 20.98, SD 49.23). Refer to for pre/postintervention differences in negative cognitions and overall emotional valence.

**Figure 2.**
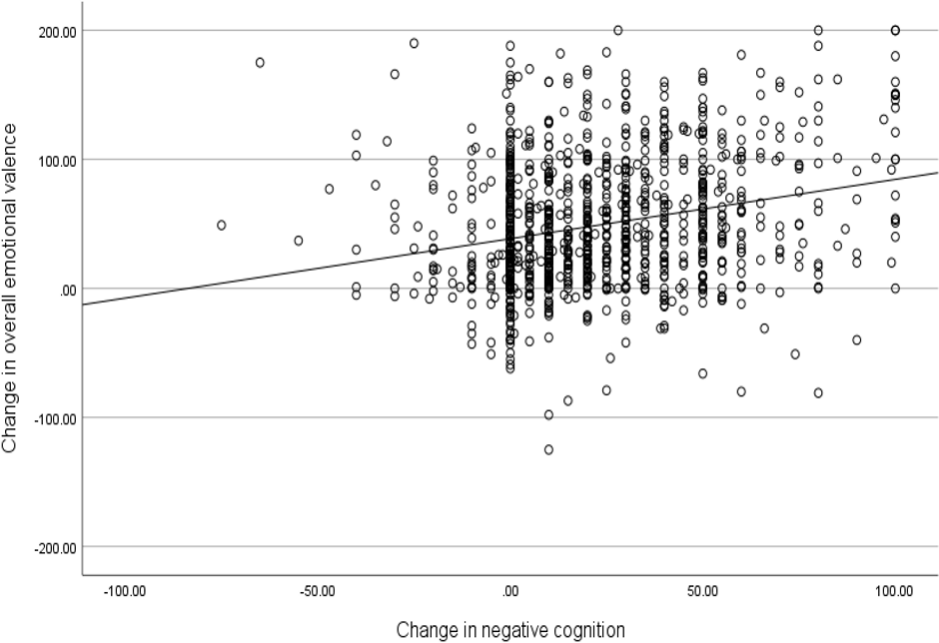
Correlation between change in negative cognition and overall emotional valence. Negative cognition change scores were computed by subtracting postscores from prescores, such that higher values indicate a reduction in the strength of belief in negative cognitions. Overall emotional valence change scores were computed by subtracting prescores from postscores, such that higher values indicate an increase in positive emotions. A positive correlation between these 2 change scores suggests that a reduction in the strength of belief in negative cognitions is associated with more positive emotions.

Given that a change in negative cognition is theorized to cause an improvement in emotional state, we tested whether the change in negative cognition (computed by subtracting postscores from prescores, such that higher values indicate a reduction in the strength of belief in negative cognitions) was correlated with the change in overall emotional valence (computed by subtracting prescores from postscores, such that higher values indicate an increase in positive emotions) and found a significant correlation (*r*=0.25; *P*<.001; refer to scatterplot of this association in Figure 2). This significant association between change in negative cognition and change in overall emotional valence supports the hypothesis that reducing belief in negative cognitions could help improve one’s emotions.

### Differences in Intervention Efficacy

In addition to the main analyses, which showed support for the efficacy of the intervention, we were interested in exploring whether the intervention differed in effectiveness based on adolescents’ demographics (ie, gender identity, race or ethnicity, SES, and grade level) as well as whether there was differential effectiveness based on how much adolescents wrote in response to the intervention thought challenge questions.

**Figure 3.**
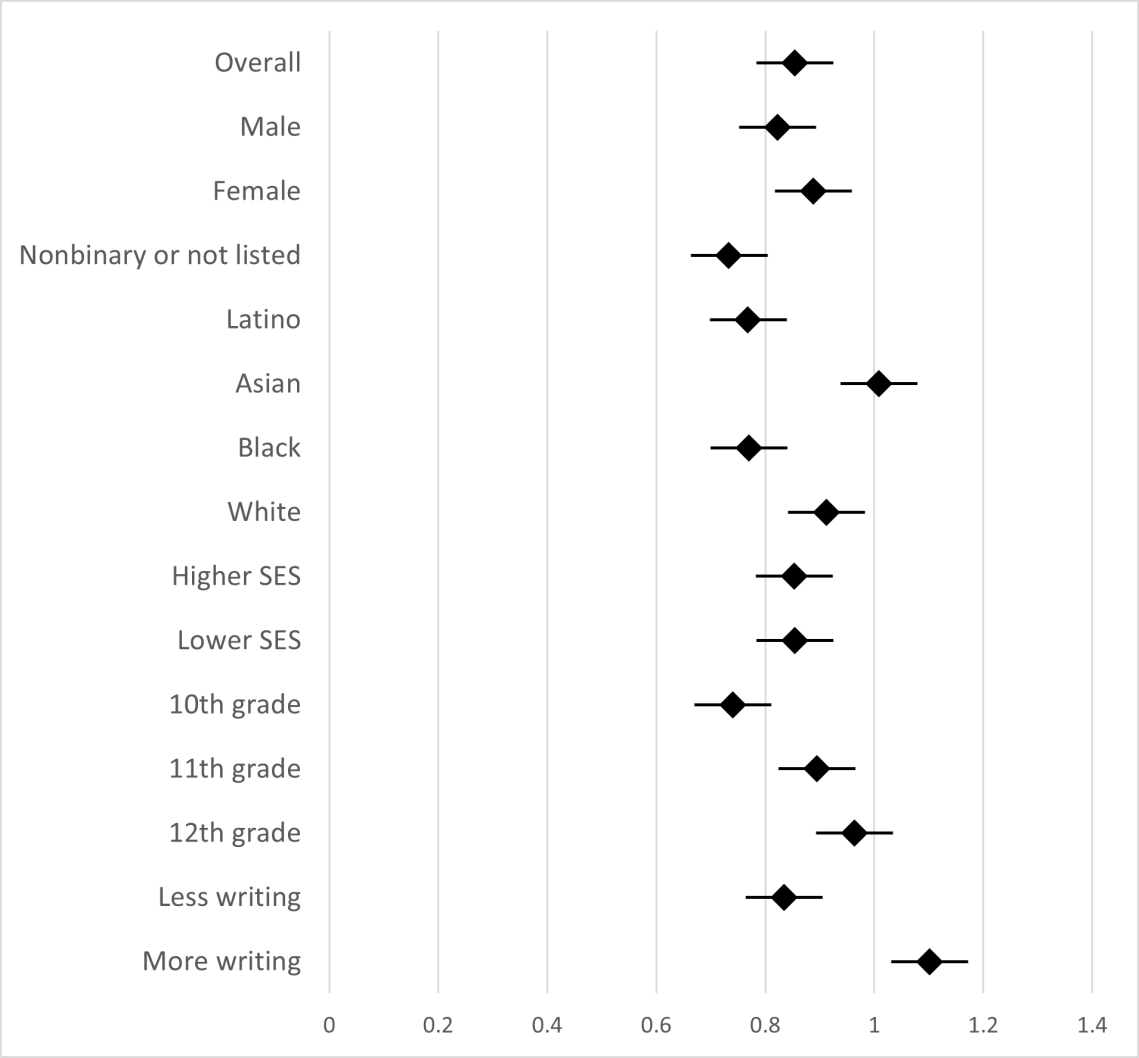
Pre/post thought record effect sizes for change in negative cognition. Higher positive effect sizes are coded to correspond to better outcomes in these panels (ie, a decrease in negative cognitions). “More” or “less” writing was based on the number of words written in response to the thought challenge questions of the activity. For illustrative purposes, “less writing” was the bottom 20% of word count and “more writing” was defined as the top 20% of word count. SES: socioeconomic status.

We ran ANCOVAs to test for differences based on adolescent gender identity, racial or ethnic identity, and SES (based on family income) on changes in negative cognition and overall emotional valence. Figures3  4 show forest plots of the effect sizes (Cohen *d*) for pre/post differences in negative cognitions and overall emotional valence. We found no differences based on adolescents’ gender identity (ie, male, female, or nonbinary) on either change in negative cognitions (*F*_2,1031_=0.87; *P*=.42) or change in overall emotional valence (*F*_2,1031_=.87; *P*=.20). For race or ethnicity group analysis, we focused on the 4 racial or ethnic groups with large enough groups of adolescents to analyze: Black, Latino, Asian, and White racial or ethnic groups. We found no differences based on adolescents’ racial or ethnic identity on either change in negative cognition (*F*_3,956_=1.18; *P*=.32) or change in overall emotional valence (*F*_3,956_=0.52; *P*=.67). We found no differences based on adolescents’ SES on either change in negative cognition (*F*_1,1027_=1.38; *P*=.24) or change in overall emotional valence (*F*_1,1027_=0.77; *P*=.38). These results suggest that the intervention was similarly effective across gender, racial or ethnic, and SES adolescent groups.

We found significant differences based on adolescents’ grade level in school on both change in negative cognition (*F*_2,1048_=6.03; *P*=.002) and change in overall emotional valence (*F*_2,1048_=3.74; *P*=.02). Post hoc least significant difference tests showed that there was less change in negative cognition and overall emotional valence for tenth graders (mean 47.61, SD 25.58; mean 16.12, SD 43.88; these means are predicted values found when controlling for preintervention measures) than for eleventh (mean 43.07, SD 25.55; mean 23.22, SD 43.88) and twelfth graders (mean 41.03, SD 25.52; mean 24.44, SD 43.68), with the only significant differences being between tenth graders compared to eleventh and twelfth graders on negative cognitions (10th-11th grade comparison: *P*=.02; 10th-12th grade comparison: *P*=.001; and 11th-12th grade comparison: *P*=.31) and overall emotional valence (10th-11th grade comparison: *P*=.03; 10th-12th grade comparison: *P*=.01; and 11th-12th grade comparison: *P*=.72). These results show that although the intervention was effective for younger adolescents (tenth graders), it was even more effective for older adolescents (eleventh and twelfth graders).

Finally, we tested whether word count for the thought challenge questions was a significant predictor of thought record efficacy with multiple regression analysis with postnegative cognition regressed on word count and prenegative cognition, and postoverall emotional valence regressed on word count and preoverall emotional valence. Results showed that word count was not a significant predictor of change in negative cognition (*F*_2,1049_=0.18; *P*=.67; *β*=−.01) or overall emotional valence (*F*_2,1049_=0.46; *P*=.50; *β*=−.02). These results suggest that adolescents benefited from the thought record similarly whether they wrote more or fewer words in response to the thought challenge questions.

**Figure 4.**
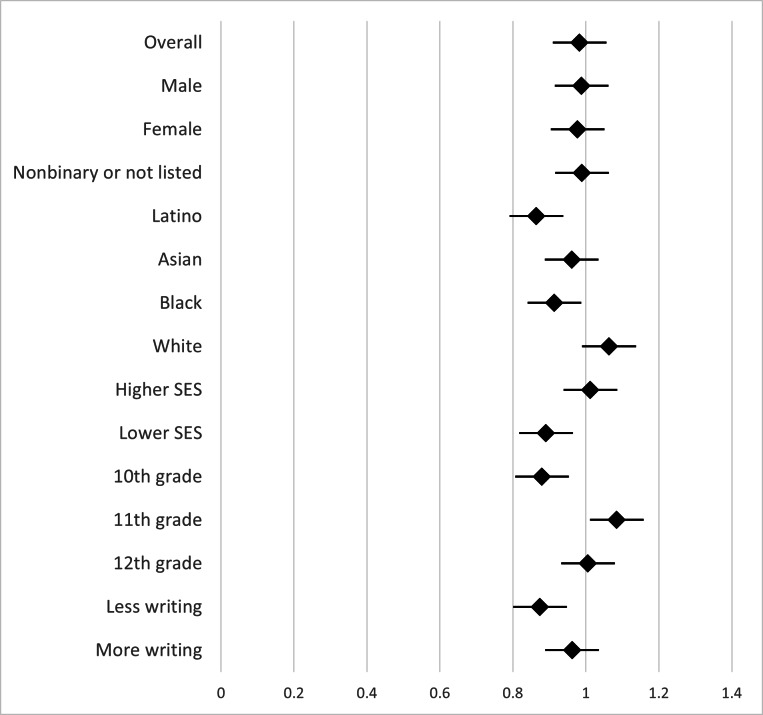
Pre/post thought record effect sizes for change in overall emotional valence. Higher positive effect sizes are coded to correspond to better outcomes in these panels (ie, an increase in positive emotions). “More” or “less” writing was based on the number of words written in response to the thought challenge questions in the activity. For illustrative purposes, “less writing” was defined as the bottom 20% of word count and “more writing” was defined as the top 20% of word count. SES: socioeconomic status.

## Discussion

### Principal Findings

The results of this study provide valuable insights into the potential of Project RE-THINK (Remember to Evaluate your thoughts: Are they True, Helpful, Inconsistent, Narrow-minded, or Knowledge-based?), a school-based digital thought record SSI, to impact the cognitive and emotional well-being of adolescents. In light of the pressing public health concerns associated with adolescent mental health [1-5], these findings support the larger goal of using brief, accessible, and effective interventions to improve adolescent outcomes. As expected, adolescents who completed Project RE-THINK reported reduced belief in their self-identified negative cognition and increased overall emotional valence (ie, an increase from negative to significantly more positive emotions). In addition, there was a significant association between improvement in negative cognition and increased positive emotional valence.

When examining group differences to better understand who this intervention may benefit, we observed no significant differences across gender identity, race or ethnicity, or SES. Although these findings suggest potential broad applicability, they should be interpreted cautiously, as nonsignificant results do not confirm equal effectiveness across all groups. There were slight differences in intervention efficacy across grade levels, with younger adolescents (tenth graders) showing improvements in both negative cognition and overall emotional valence, but older adolescents (ie, eleventh and twelfth graders) showing even bigger improvements in both negative cognition and overall emotional valence. In addition, the total amount written in response to the thought challenge questions was not significantly associated with any outcomes, suggesting that adolescents benefited whether they wrote longer or shorter responses.

Overall, the Project RE-THINK intervention had positive and expected results, showing support for its utility as an SSI in a classroom setting. Furthermore, because we were able to test this intervention in a large, diverse group of adolescents, we were able to assess its broad accessibility and efficacy among a community rather than a convenience sample. Future research should continue to test this intervention with even larger and even more diverse samples to further assess whether effectiveness varies across contexts and demographic groups, given that some school-based psychological interventions show similar efficacy across students and school contexts [35], whereas others show important contextual and student variation [43]. In addition, future studies should explore whether the intervention’s effectiveness differs based on the severity of the initial distressing situation. Examining whether adolescents with more intense negative cognitions or emotional distress benefit similarly to those experiencing moderately upsetting situations could provide valuable insight into the intervention’s range and applicability.

There are few studies using SSIs in classrooms at a large scale, and these results support Project RE-THINK as a potentially helpful prevention and intervention strategy. School-based interventions have the potential to play an important role in supporting adolescents’ mental health. This intervention is brief, can be provided broadly to adolescents, and reduces barriers to mental health programs that are otherwise difficult to engage. SSIs can also reduce some of the burden on schools that do not have enough full-time mental health professionals by giving adolescents tools to manage certain challenging thoughts and emotions. School-based interventions can be done each year or at higher frequencies, as schools determine what makes sense for their students. Given that some data suggest that SSIs can be as effective as other longer-term psychotherapy [41], school-based SSIs may be an important avenue for dissemination, implementation, and scaling interventions at low cost.

Project RE-THINK had an important impact on negative cognitions and overall emotional valence. As predicted, there was a decrease in negative cognition beliefs and an increase in overall positive emotional valence, which would be expected given the target of the intervention [26]. Adolescents who engaged with this SSI experienced a marked reduction in their belief in self-identified negative cognitions, indicating that the intervention effectively targeted and altered these maladaptive thought patterns. Simultaneously, participants exhibited a significant increase in their overall emotional valence, signifying a notable shift from predominantly negative emotions to a significantly more positive emotional state. The correlation between the improvement in negative cognition beliefs and the increase in overall positive emotional valence underscores the critical relationship between cognitive restructuring and changing emotions that has been both observed and theorized in CBT [44].

Changing negative cognitions to be less negative and more realistic and balanced is a transdiagnostic treatment used for a variety of mental health issues [25]. Similarly, decreasing negative emotion, often driven by a change in negative thoughts, is also an important target across CBTs. Project RE-THINK provides an intervention that is not only targeted toward one type of mental health disorder, but rather, it is a transdiagnostic mechanism that can more universally be applied across individuals. This point is important, as Project RE-THINK can best be conceptualized as both a prevention strategy and an intervention for those who may be struggling across stressors or potential clinical diagnoses.

Our exploration of potential group differences shed light on the effectiveness of Project RE-THINK for different demographic groups. The findings revealed that the intervention’s impact was consistent across gender identity, race or ethnicity, and SES, underscoring the intervention’s potential for broad applicability. As mentioned, the transdiagnostic approach to Project RE-THINK may be part of the reason for these results. Conversely, other SSIs have had differing effects among a variety of groups (for overview refer to [38]). Although our intervention was generally helpful across groups, we note that more tailored and targeted interventions for specific groups and specific issues are equally important to develop and implement. Choosing an SSI requires careful consideration among key people and interested groups. Future research should consider what intervention should be used, in what situation, for whom, and how often to use SSIs.

In testing for group differences, we found that although younger adolescents benefited from the intervention, older adolescents exhibited even more substantial improvements in both negative cognition beliefs and overall emotional valence. This finding suggests that the intervention’s efficacy may increase with age, potentially due to older adolescents’ more advanced cognitive development, emotion regulation skills, or life experiences. Specifically, older adolescents may have more experience using emotion regulation and cognitive restructuring skills, and these skills may also be easier for them to implement.

The finding that the total amount written during the intervention was not associated with the outcomes is also important to note. This suggests that completing the intervention, rather than the volume of written content, was sufficient to elicit the observed changes in cognitive and emotional states. This simplicity and flexibility in the engagement process might enhance the intervention’s appeal and accessibility to a broader audience.

### Limitations and Future Directions

This study has several strengths (eg, large sample size, use of an evidence-based intervention, and the flexibility and scalability of the SSI); however, there are also important limitations that support future research in this area. First, the outcome measures were immediate outcomes and focused on an underlying mechanism related to psychopathology (ie, negative cognition) and overall emotional valence change from pre- to postintervention. These measures are aligned with other measures in thought record research [21-24,29], but do not represent a fuller range of measures or long-term measures. Adding additional outcomes, such as general psychopathology symptoms and a longer follow-up period, would provide a better understanding of the intervention’s impact and trajectory and bolster the evidence for its broader impacts. A second limitation is the quasi-experimental design, which may introduce expectancy effects, where participants respond based on perceived intervention goals rather than actual cognitive or emotional changes. However, as the activity was integrated into regular classwork, this may have reduced pressure to respond in socially desirable ways. Third, the intervention was done only once during the study period. Given that in CBT, individuals practice skills over time and receive feedback, there may be important work to determine what the right dose of this type of intervention is for adolescents in order to have the largest and longest impact. Although adolescents in our study showed promising results, being able to translate these skills across stressors and situations as well as practice the skill over time may be critical for skill development and utility. Future research should consider the use of SSIs more than once to develop mastery of the skill and the impact of using multiple and different SSIs to build skills. Fourth, this intervention could provide insight through understanding the ways individuals write during this task. For example, some research has shown that those who write about their emotional situations with greater linguistic distance (eg, write about it in the past tense or use third-person pronouns) may have more effective outcomes [45]. Examining the content of the writing may provide critical insight into both how the intervention works and how to maximize the impact of the intervention. These important questions can lead to a better understanding of SSIs and how to maximize their impact. Fifth, this study was conducted within a single school, which may limit the generalizability of the findings, and future research should continue toward replicating these findings across schools and student populations.

Future studies may consider using a randomized controlled trial design to examine causality, as this study was quasi-experimental, which limits causal inferences about the intervention’s effects. Future studies may look to examine a comparison of this SSI to a variety of control groups and potentially a comparison with other SSIs that have been tested in this age group. In addition, although the sample size was large and included a diverse group of individuals, future research should focus on casting an even larger and wider net of potential adolescents to help determine if the effects replicate across contexts. Given the rich nature of the written responses generated by the intervention, future research could explore how linguistic patterns relate to intervention effectiveness. This is the focus of ongoing work examining how writing characteristics may be linked to cognitive and emotional outcomes [45]. Finally, the utility of a digital SSI is greatly impacted by who has access to the intervention. Although school-based interventions may enhance accessibility through their structure and format, further research is needed to assess whether this SSI can be effectively disseminated across diverse settings and formats to increase uptake and impact. It is also important to foster autonomy and authentic student engagement for mandatory activities in school settings, as previous intervention research has demonstrated [46].

### Conclusions

Overall, this study tested the Project RE-THINK intervention, a brief digital thought record and CBT-based intervention for adolescents, in a school setting. By providing adolescents with tools to recognize, challenge, and restructure their negative cognitions, Project RE-THINK has the potential to equip them with skills that could extend beyond the single session and be used as a prevention program in addition to the immediate benefits it showed in this study. These skills could be applied to various life situations, offering adolescents a valuable resource for managing and preventing emotional difficulties. Among the adolescents who engaged in the SSI, individuals generally saw positive outcomes with large immediate effects on both reducing negative cognitions and improving overall positive emotional valence. These pre/post intervention effects provide initial support that RE-THINK is a useful intervention for many adolescents. Using a school-based intervention framework, RE-THINK was able to reach adolescents who may not have access to, or who may not typically engage in, this type of intervention. Given the vast need for mental health prevention and intervention programs that continue to grow, using low-intensity SSIs in schools may provide an avenue that supports adolescents’ needs while supporting the already taxed mental health support system.

## Abbreviations

CBT: cognitive-behavioral therapy
RE-THINK: Remember to Evaluate your thoughts: Are they True, Helpful, Inconsistent, Narrow-minded, or Knowledge-based?
SES: socioeconomic status
SSI: single-session intervention
